# Absence seizures with intellectual disability as a phenotype of the 15q13.3 microdeletion syndrome

**DOI:** 10.1111/j.1528-1167.2011.03301.x

**Published:** 2011-12

**Authors:** Hiltrud Muhle, Heather C Mefford, Tanja Obermeier, Sarah von Spiczak, Evan E Eichler, Ulrich Stephani, Thomas Sander, Ingo Helbig

**Affiliations:** * Department of Neuropediatrics, University Medical Center Schleswig-Holstein, Christian-Albrechts UniversityKiel, Germany; † Division of Genetic Medicine, Department of Pediatrics, University of WashingtonSeattle, Washington, U.S.A.; ‡ Department of Genome Sciences and Howard Hughes Medical Institute, University of WashingtonSeattle, Washington, U.S.A.; § Cologne Center for Genomics, University of CologneCologne, Germany

**Keywords:** Intellectual disability, Idiopathic generalized epilepsy

## Abstract

15q13.3 microdeletions are the most common genetic findings identified in idiopathic generalized epilepsies to date, and they are present in up to 1% of patients. In addition, 15q13.3 microdeletions have been described in patients with epilepsy as part of a complex neurodevelopmental phenotype. We analyzed a cohort of 570 patients with various pediatric epilepsies for 15q13.3 microdeletions. Screening was performed using quantitative polymerase chain reaction; deletions were confirmed by array comparative genomic hybridization (CGH). We carried out detailed phenotyping of deletion carriers. In total, we identified four pediatric patients with 15q13.3 microdeletions, including one previously described patient. Two of four deletions were de novo, one deletion was inherited from an unaffected parent, and for one patient the inheritance is unknown. All four patients had absence epilepsy with various degrees of intellectual disability. We suggest that absence epilepsy accompanied by intellectual disability may represent a common phenotype of the 15q13.3 microdeletion in pediatric patients with epilepsy.

Structural genomic variations are increasingly recognized as genetic risk factors for various epilepsies (Mefford et al., 2010). Among the different microdeletions described to date, 15q13.3 stands out as the most common genetic risk factor for epilepsy. Specifically, 15q13.3 microdeletions are found in up to 1% of patients with idiopathic generalized epilepsy (IGE) (Dibbens et al., 2009; Helbig et al., 2009), but have also been described in other types of seizure disorders. Although15q13.3 microdeletions also predispose to a wide spectrum of other neurodevelopmental disorders including intellectual disability, autism, and schizophrenia (Sharp et al., 2008; Stefansson et al., 2008; van Bon et al., 2009), epilepsy appears to be a frequent feature in deletion carriers.

Given the broad phenotype associated with 15q13.3 microdeletions, we screened a pediatric epilepsy cohort for 15q13.3 microdeletions, including patients with various degrees of intellectual disability.

## Methods

### Clinical analysis

A cohort of 570 children and adolescents with various epilepsy subtypes, febrile seizures, and epilepsy-related electroencephalography (EEG) features (Table S1) were included, and iterative phenotyping (Manolio et al., 2009) was performed in individuals with identified deletions. The majority of patients were Caucasian. None of the 570 patients had been included in previous studies (Helbig et al., 2009; Mefford et al., 2010). Given the phenotypic features, one previously described patient (Helbig et al., 2009; ID 1674) was included. This patient was not included in the statistical analysis.

Detailed phenotypic analysis and review of multiple EEG studies was performed in three of four patients. Phenotyping was carried out in family members, if available; the family of proband 3 was lost to follow-up. Epileptic syndromes were described according to the classifications of the International League Against Epilepsy (Engel, 2001). Written informed consent was obtained by participants or parents in the case of minors. The study was approved by the local ethics.

### Genetic analysis

Screening of the cohort for 15q13.3 microdeletions was performed using quantitative polymerase chain reaction (PCR; TaqMan®; Applied Biosystems, Foster City, CA, U.S.A.), with intronic probes for the second intron of *CHRNA7*. Confirmation of 15q13.3 microdeletions and refining of the breakpoints in probands and screening of family members was performed using high-resolution custom array comparative genomic hybridization (CGH) with 4,398 probes in the 15q13 BP3-BP5 region (hg18, chr15:26,500,000–31,000,000; average probe spacing 1,250 bp; Agilent Technologies, Santa Clara, CA, U.S.A.).

## Results

In a cohort of 570 pediatric patients, we identified three individuals with a 15q13.3 microdeletion. All three individuals as well as a previously described patient presented with various degrees of intellectual disability and absence epilepsy. Dysmorphic features were not present. Clinical characteristics of the probands and their families are summarized in Tables S1 and S2.

The 15q13.3 microdeletion was found exclusively in patients with generalized epilepsies (3 of 101 IGE patients) compared to the remaining pediatric patients predominantly with focal epilepsies and febrile seizure–related syndromes (0 of 469). This difference was statistically significant (p = 0.006). In addition, the microdeletion was present exclusively in patients with absence seizures (3 of 31 vs. 0 of 539; p = 0.0002). None of the deletion carriers had additional chromosomal rearrangements.

### Proband 1

The girl was born at term to nonconsanguineous healthy parents of Northern European ancestry. Birth parameters, postnatal period, and acquisition of motor skills were normal; cognitive and language development was delayed.

**Figure 1:**
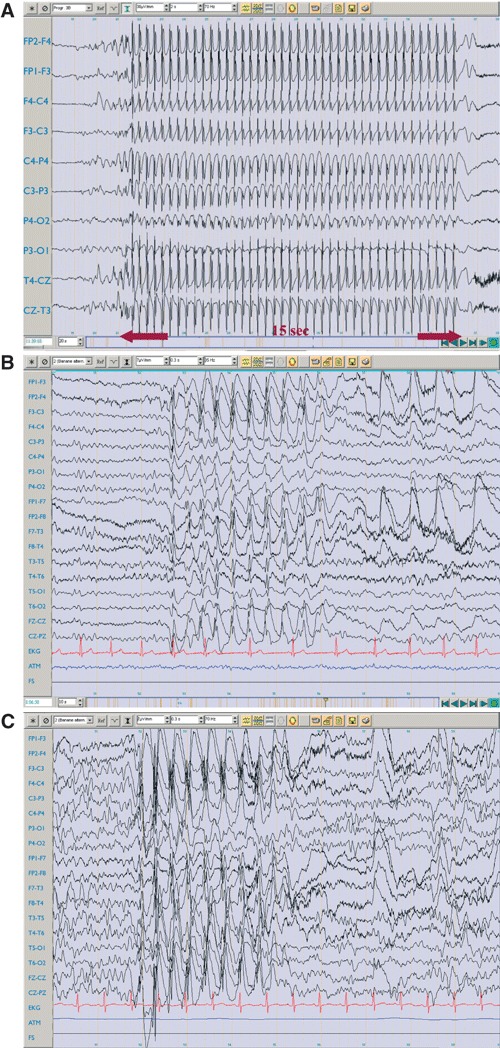
EEG recordings of 15q13.3 microdeletion carriers. (A) Bilateral synchronous regular 3-Hz spike-wave discharges in proband 1 lasting for 15 s associated with clinical absence seizures. (B) Absence seizure in proband 2 lasting for 4 s. (C) Spike-wave discharges lasting for 3 s in proband 4. This proband was affected by absence status lasting for >6 h.

At the age of five, the patient presented with severe intellectual disability, autistic features, and typical absence seizures. The EEG showed paroxysms of regular 3 Hz generalized spike-waves lasting for up to 15 s, accompanied by clinical absences (Fig. 1A). Seizures were controlled on ethosuximide, which was withdrawn at the age of eight. Relapse of absence seizures occurred in puberty, again responsive to ethosuximide. At the age of 14, EEG recordings showed photosensitivity; at the age of 19, the EEG and magnetic resonance imaging (MRI) were normal.

**Figure 2:**
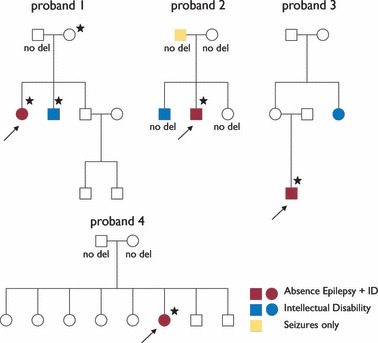
Pedigrees of 15q13.3 microdeletion carriers. All four probands (marked by the arrow) are affected by absence seizures and intellectual disability. Additional microdeletion carriers (family of proband 1) are marked by an asterisk. No deletions (“no del”) were found in several relatives investigated by array-CGH.

Her brother presented with severe intellectual disability, but not epilepsy. Both parents and a second brother were unaffected (Fig. 2). The EEG of the mother (unaffected deletion carrier) showed background slowing (7 Hz) and no epileptic discharges. Microdeletions of 15q13.3 between breakpoints BP4 and BP5 were detected in the proband (Fig. S1), the brother with intellectual disability, and the unaffected mother.

### Proband 2

The boy was born at term after an unremarkable pregnancy with normal birth parameters as the second child of nonconsanguineous parents of Turkish ancestry. Early motor and cognitive development was unremarkable.

Frequent absence seizures were noted at the age of 7 accompanied by regular generalized 3-Hz spike-wave discharges. Mild intellectual disability became obvious at the age of 8. Valproic acid therapy suppressed absence seizures, which relapsed twice upon discontinuation at the ages of 12 and 16 (Fig. 1B). A single generalized tonic–clonic seizure (GTCS) occurred in the context of noncompliance during adolescence. At the age of 24, occasional absences are reported. The proband has attended a school for intellectually disabled children and aggressive behavior is reported.

The proband’s brother (26 years) has moderate intellectual disability. The father had uncharacterized epileptic seizures in childhood with normal intellectual ability. The proband’s sister (18 years) and mother are unaffected. A de novo microdeletion of 15q13.3 was detected between breakpoints BP4 and BP5.

### Proband 3

The boy is the only child of unrelated healthy parents of Northern European descent. Pregnancy, birth parameters, and postnatal period were unremarkable.

The proband presented with rare typical absence seizures starting at the age of 13, consistent with juvenile absence epilepsy. Seizures were controlled on ethosuximide. EEG recordings were not available and the family was lost for follow-up. The patient is attending a school for intellectually disabled children. Aggressive behavior was reported. A maternal aunt was reported to have intellectual disability with unknown etiology.

A 15q13.3 microdeletion between breakpoints BP4 and BP5 was identified in the proband. Family members were not available for testing.

### Proband 4

The girl was born at term as the sixth child to nonconsanguineous parents of Northern European ancestry. Birth parameters and postnatal period were unremarkable. Gross and fine motor skills and language development were unremarkable in the first 4 years of life. Learning difficulties became obvious in childhood and the patient attended a school for intellectually disabled children.

At the age of 17, the proband presented with a single GTCS with subsequent stupor lasting for >6 h. The EEG revealed continuous spike-wave discharges consistent with absence status, which was interrupted by intravenous clonazepam. In addition, the patient had brief 3-Hz generalized spike-wave discharges (Fig. 1C). MRI imaging revealed a small right occipital subependymal periventricular heterotopia (Fig. S2). The patient became seizure-free on lamotrigine and topiramate.

Family history is negative for seizures or intellectual disability. A de novo 15q13.3 microdeletion was identified in the proband.

## Discussion

In our study of a pediatric epilepsy cohort, we identified four patients altogether with 15q13.3 microdeletions presenting with absence seizures accompanied by intellectual disability.

Some children with typical absence epilepsy may have learning disabilities (Titomanlio et al., 2007; Wakamoto, 2008), but the frequency of the combination of typical absence epilepsy and obvious intellectual disability is unknown. Interestingly, this combination of phenotypes was found in previous studies in 15q13.3 microdeletion carriers (Sharp et al., 2008; Helbig et al., 2009).

Given that our cohort consisted of unselected cases with pediatric epilepsies, the similarity of the phenotypes of 15q13.3 microdeletion carriers was surprising. All patients had absence seizures and various degrees of intellectual disability. The association of 15q13.3 microdeletions with absence epilepsy compared to other phenotypes seen in our cohort was statistically significant. The similarity of the phenotypes in our pediatric cohort is contrasted by the otherwise heterogeneous phenotypes associated with this microdeletion, including a broad spectrum of neuropsychiatric and neurologic disorders.

The phenotypes associated with the 15q13.3 microdeletion depend on the cohort investigated. Particularly, inclusion and exclusion criteria such as intellectual disability in epilepsy cohorts can influence the findings. For example, intellectual disability is a typical exclusion criterion for IGE, a common 15q13.3 microdeletion-related phenotype.

We suggest the following explanation for the similar phenotypes in our cohort: Because the 15q13.3 microdeletion is known to increase risk for both absence epilepsy and intellectual disability, the frequency of this variant is likely to be increased in “overlap phenotypes.”

The variable phenotypic expression of the 15q13.3 microdeletion families 1–2 is intriguing. In family 1, the variant is inherited from an apparently unaffected mother. In family 2, the proband’s father is affected by epilepsy and the proband’s brother has intellectual disability. However, the variant is present only in the proband. Incomplete segregation and the existence of affected family members not carrying the variant has been observed previously in families with 15q13.3 microdeletions (Dibbens et al., 2009) and is as yet unexplained. Subtle unobserved neuropsychological phenotypes in apparently unaffected carriers and the existence of strong modifier genes in families might account for some of this variability.

## Conclusion

Awareness of the associated phenotypes should alert clinicians to consider 15q13.3 microdeletions. In particular the comorbidity of absence seizures and intellectual disability should prompt screening for this particular chromosomal imbalance, which may represent a common constellation for the 15q13.3 microdeletion in patients with pediatric epilepsy.

## Supporting Information

Figure S1 Array-CGH in probands with identified 15q13 microdeletions.

Figure S2 MRI scans of patient 4 demonstrating a small right occipital subependymal periventricular heterotopia marked by an arrow.

Table S1 Phenotypes of probands screened for 15q13.3 microdeletions.

Table S2. Clinical and genetic data in 15q13.3 microdeletion carriers.
