# Supplementary figures and images for: Absence seizures with intellectual disability as a phenotype of the 15q13.3 microdeletion syndrome
Source: Epilepsia. 2011 Dec;52(12):e194–8. doi: 10.1111/j.1528-1167.2011.03301.x (PMC3270691; doi:10.1111/j.1528-1167.2011.03301.x)

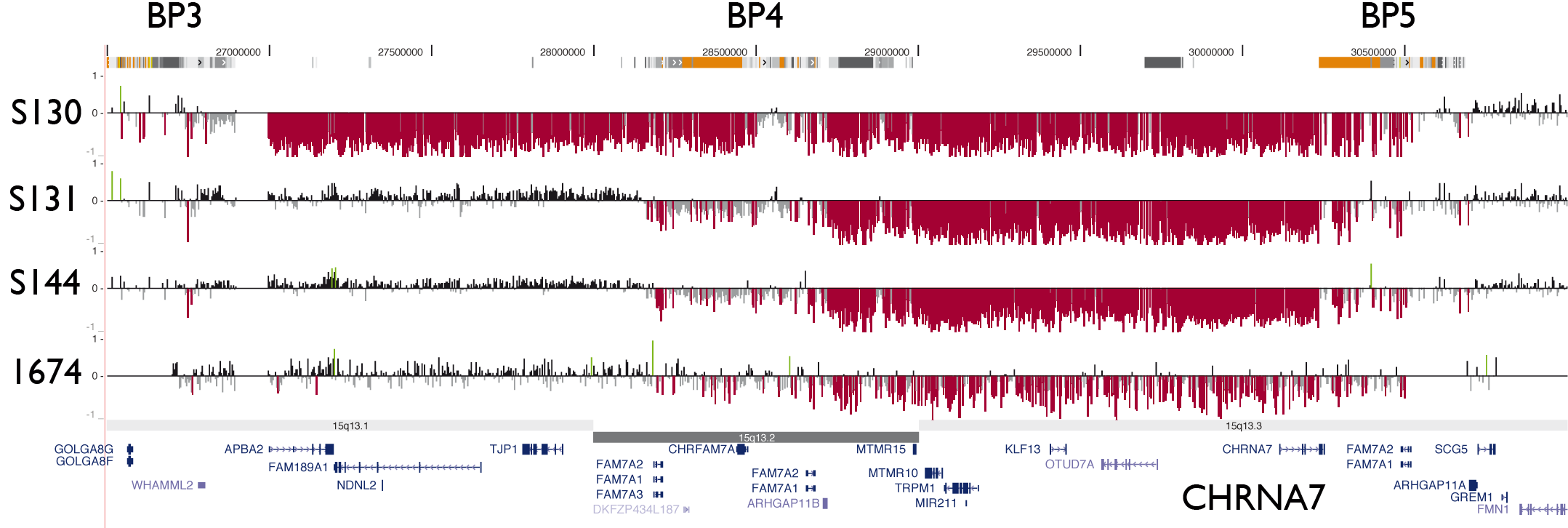

Supplement: Figure S1 — Array-CGH in probands with identified 15q13 microdeletions. [file epi0052-e194-SD1.tif]

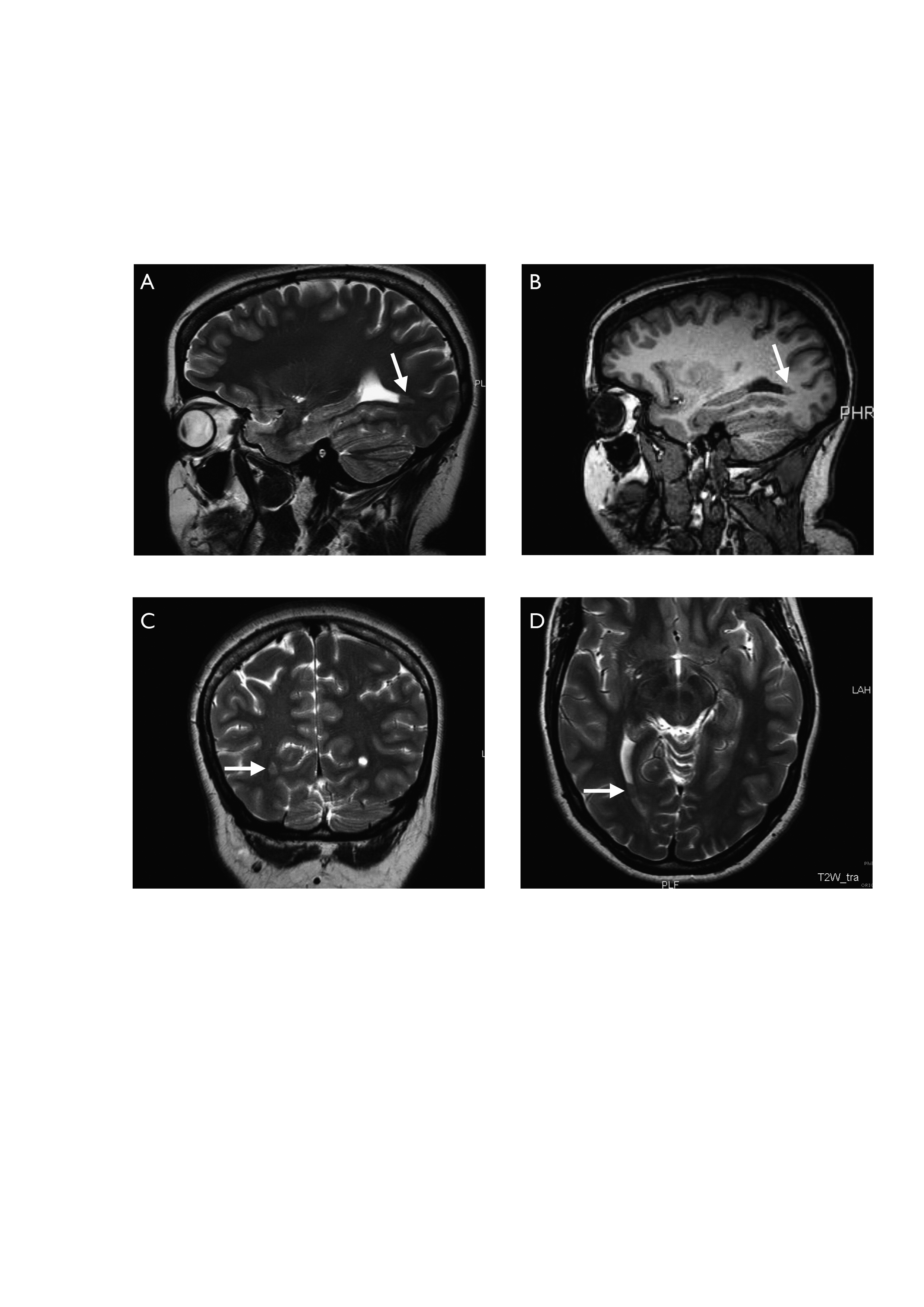

Supplement: Figure S2 — MRI scans of patient 4 demonstrating a small right occipital subependymal periventricular heterotopia marked by an arrow. [file epi0052-e194-SD2.tif]
