# Supplementary material for: Absence seizures with intellectual disability as a phenotype of the 15q13.3 microdeletion syndrome
Source: Epilepsia. 2011 Dec;52(12):e194–8. doi: 10.1111/j.1528-1167.2011.03301.x (PMC3270691; doi:10.1111/j.1528-1167.2011.03301.x)
Supplement: Supplementary file 3 [file epi0052-e194-SD3.doc]

**Supplemental Figure 1. Array-CGH in probands with identified 15q13 microdeletions** Proband 1 (1674) was previously described by (Helbig, et al. 2009). Three additional carriers were detected by screening a pediatric cohort of 570 children with epilepsy or seizures (Proband 2 = S 131, proband 3 = S 144, proband 4 = S 130). All probands are affected by absence epilepsy and various degrees of intellectual disability. For each individual, deviations of probe log_2_ ratios from zero are shown in grey and black. Log_2_ ratios exceeding a threshold of 1.5 are shown in red, indicative of a deletion. Segmental duplication are shown as yellow and orange bars, overlapping with the genetic breakpoints (BP3, BP4 and BP5) on chromosome 15.

**Supplemental Figure 2.** MRI-scans of patient 4 demonstrating a small right occipital subependymal periventricular heterotopia marked by an arrow.

**Supplemental Table 1. Phenotypes of probands screened for 15q13.3 microdeletions.**

**Supplemental Table 2. Clinical and genetic data in 15q13.3 microdeletion carriers**
