# Supplementary material for: Absence seizures with intellectual disability as a phenotype of the 15q13.3 microdeletion syndrome
Source: Epilepsia. 2011 Dec;52(12):e194–8. doi: 10.1111/j.1528-1167.2011.03301.x (PMC3270691; doi:10.1111/j.1528-1167.2011.03301.x)
Supplement: Table S1 — Phenotypes of probands screened for 15q13.3 microdeletions. [file epi0052-e194-SD4.doc]

**Supplemental Table 1. Phenotypes of probands screened for 15q13.3 microdeletions**

| Classification of pediatric probands | Intellectual  disability | N = 570 |
| --- | --- | --- |
| IGE (n = 101) |  |  |
| MAE | **3** | 5 |
| BMEI | **1** | 1 |
| EOAE | **2** | 5 |
| CAE | **2** | 19 |
| JAE | **2** | 8 |
| JME | **0** | 9 |
| GTCS alone | **1** | 45 |
| Unclassified IGE | **5** | 9 |
| Idiopathic focal epilepsies (n = 20) |  |  |
| BETCS /Panayiotopolous syndrome | **1** | 10 |
| ABPE | **5** | 10 |
| Other epilepsies (n = 201) |  |  |
| Dravet syndrome | **16** | 16 |
| Symptomatic focal epilepsies* | **45** | 74 |
| Cryptogenic focal epilepsies | **23** | 74 |
| Unclassified epilepsies | **10** | 37 |
| Fever associated epilepsy syndromes (n = 126) |  |  |
| FS alone | **2** | 102 |
| FS plus (additional afebrile seizures in patient) | **1** | 10 |
| GEFS plus (additional afebrile seizures in relatives) | **4** | 14 |
| EEG abnormalities, unclassified single seizures (n = 122) |  |  |
| Unclassified single seizures | **2** | 12 |
| Generalized spike-wave, focal sharp-wave (no seizures) | **1** | 14 |
| **Photoparoxysmal response (no seizures)** | 5 | 96 |

IGE, idiopathic generalized epilepsy; EOAE, early onset absence epilepsy; CAE, childhood absence epilepsy; JAE, juvenile absence epilepsy; AE, absence epilepsy; JME, juvenile myoclonic epilepsy; BMEI, benign myoclonic epilepsy of infancy; MAE, myoclonic astatic epilepsy; GTCS, generalized tonic clonic seizure; BETCS, benign epilepsy with centrotemporal spikes; ABPE, atypical benign partial epilepsy (Pseudo-Lennox syndrome); FS, febrile seizures; GEFS plus, generalized (genetic) epilepsy with febrile seizures plus; * including symptomatic West-/Lennox-Gastaut syndrome.
