# Supplementary material for: Absence seizures with intellectual disability as a phenotype of the 15q13.3 microdeletion syndrome
Source: Epilepsia. 2011 Dec;52(12):e194–8. doi: 10.1111/j.1528-1167.2011.03301.x (PMC3270691; doi:10.1111/j.1528-1167.2011.03301.x)
Supplement: Table S2. — Clinical and genetic data in 15q13.3 microdeletion carriers. [file epi0052-e194-SD5.doc]

**Supplemental Table 2. Clinical and genetic data in 15q13.3 microdeletion carriers**

|  | **Proband 1** | **Proband 2** | **Proband 3** | **Proband 4** |
| --- | --- | --- | --- | --- |
| **Gender** | female | male | male | female |
| **Ethnic origin** | Northern European | Mediterranean | Northern European | Northern European |
| **15q13.3 microdeletion** | BP4-BP5 | BP4-BP5 | BP4-BP5 | BP3-BP5 |
| **De novo vs. inherited**  **Microdeletions** | del in mother  del in brother | *de novo* | unknown | *de novo* |
| **Family history of :**  **-Seizures**  **-Intellectual disability** | No  Yes | Yes  Yes | No  Yes | No  No |
| **Age of onset**  **first seizure** | 5 years  Absences  (>10 /day) | 7 years  Absences  (>10 / day) | 13 years  Absences | 17 years  Absence status |
| **Age at last**  **follow up** | 19 years | 21 years | 16 years | 21 years |
| **Additional seizure type** | None | GTCS | None | GTCS |
| **Epilepsy syndrome** | Resemble CAE, but intellectual disability | CAE transitioning to JAE | JAE | JAE |
| **Degree of intellectual disability** | Severe | Moderate | Mild | Mild |
| **Behavior**  **abnormalities** | Autistic features | Aggressive behaviour | Aggressive behaviour | None |
| **EEG**  **Sleep EEG** | 3 Hz sw  PPR IV  Normal background  (8-9 Hz)  Dysrhythmic groups  Abortive sw | 3 Hz sw  Normal background  (9 Hz)  Regular and irregular sw | 3 Hz sw  PPR IV  Normal background  (9 Hz)  Not performed | 2,5-4 Hz sw  irregular sw  Normal background  (9-10 Hz)  Not performed |
| **MRI brain** | Normal | Not performed | Not performed | Right occipital PVNH |

**Abbreviations:** CAE = Childhood Absence Epilepsy, JAE = Juvenile Absence Epilepsy; GTCS = Generalized Tonic Clonic Seizure; Hz = Herz; sw = spike wave discharges; PPR = Photoparoxysmal Response, PVNH = periventricular nodular heterotopia
